# Personalized Sliding Window Recommendation Algorithm Based on Sequence Alignment

**DOI:** 10.3390/e24111662

**Published:** 2022-11-15

**Authors:** Lei Zhou, Bolun Chen, Hu Liu, Liuyang Wang

**Affiliations:** 1 Faculty of Computer and Software Engineering, Huaiyin Institute of Technology, Huaian 223003, China; 2 Department of Physics, University of Fribourg, CH-1700 Fribourg, Switzerland

**Keywords:** personalized sliding window, recommendation algorithm, sequence alignment

## Abstract

With the explosive growth of the amount of information in social networks, the recommendation system, as an application of social networks, has attracted widespread attention in recent years on how to obtain user-interested content in massive data. At present, in the process of algorithm design of the recommending system, most methods ignore structural relationships between users. Therefore, in this paper, we designed a personalized sliding window for different users by combining timing information and network topology information, then extracted the information sequence of each user in the sliding window and obtained the similarity between users through sequence alignment. The algorithm only needs to extract part of the data in the original dataset, and the time series comparison shows that our method is superior to the traditional algorithm in recommendation Accuracy, Popularity, and Diversity.

## 1. Introduction

With the continuous development of electronic technology and network technology, people can obtain a variety of services and products on the Internet through computers or mobile devices. According to statistics, the number of global Internet users is 4.388 billion, and the average online time is more than 6 h per day. Of the top ten most popular websites in the world counted by Alexa, seven are social networking sites. A large number of users share their lives, watch videos, and shop on the Internet. These numerous interactions among online users constitute a complex social network. The emergence of social networks has greatly satisfied the needs of users, making communication between users more convenient, working more efficiently, and living styles richer.

However, such richness of information is not always beneficial. Even with detailed keywords, online users still find it difficult to obtain the products or services they actually want, and the process is always time-consuming. Examples are finding people you know on Facebook or finding your favorite items on shopping websites. How to help users obtain the required information accurately has become a problem that researchers have been studying for years. Researchers have developed a tool that is called the recommendation system. The recommendation system analyses the user’s interactive behaviors with their online information, such as browsing habits, preferences, and following content, and recommends content that the user may be interested in and help the user to make decisions.

As we know, user preferences are not immutable and frozen; they will change over time. In addition, the impact of other users and the environment around them is also an important factor that cannot be ignored. Traditional recommendation algorithms often ignore temporal information and the structural relationships between users. The effective use of temporal information and the connection between users can enrich users’ information and more accurately identify users’ personal interests, which has important theoretical value and practical application prospects in the application of recommendation systems. Based on this, this paper proposes a personalized sliding window recommendation algorithm combining temporal and topology information in social networks. This algorithm calculates the similarity between users according to the information sequence of different users in the sliding window and only needs to extract part of the original dataset, which can improve the execution efficiency and the accuracy of the recommended algorithm.

The remainder of this paper is structured as follows: Section 2 introduces the related works, and summarizes and compares their algorithms. Section 3 describes the recommended method based on sequence alignment proposed in this paper, and specifically introduces the algorithm idea and implementation framework. Section 4 gives the analysis and discussion of the experimental results. Section 5 will summarize the work of this paper.

## 2. Related Works

According to the recommendation strategy, recommendation algorithms can be divided into four categories, which are a recommendation algorithm based on collaborative filtering [1,2,3], content-based recommendation algorithm [3,4,5,6,7], recommendation algorithm based on demographic filtering [8], and hybrid filtering recommended algorithm [9]. Among these recommended methods, collaborative filtering is the most widely used algorithm. The basic idea of a collaborative filtering recommendation algorithm is that if users have the same interests in the past, they tend to have the same interests in the future. The collaborative filtering recommendation algorithm can deal with a large amount of data that are usually difficult to process, and it is not affected by data format while mining new points of interest of users, but there are still many problems, the most typical of which are network sparsity problems and cold start problems, these problems are more obvious in the multi-relational network.

Many scholars have carried out a lot of research to solve these problems. Lin et al. [10] present a multiobjective personalized recommendation algorithm using extreme point-guided evolutionary computation (called MOEA-EPG). Zhao et al. [11] proposed a novel Cross-Domain Collaborative Filtering (CDCF) algorithm termed the Low-rank and Sparse Cross-Domain (LSCD) recommendation algorithm. Different from the majority of the CDCF algorithms that tri-factorize the rating matrix of each domain into three low-dimensional matrices, LSCD extracted a user and an item latent feature matrix for each domain, respectively. HongXia W [12] proposed an improved collaborative filtering algorithm based on properties to improve the utilization of data resources and decrease the sparse degree of the matrix. Wu et al. [13] proposed a new algorithm based on user rating probability and project type (UPCF) to deal with the sparsity of data and found that it performed better than the conventional algorithms.

In recent years, it has been widely used to improve the performance of the collaborative filtering algorithm by using user relationships and time information.

### 2.1. Collaborative Filtering Algorithm Based on User Relationships Mining

Many researchers successfully used user relationship information to improve collaborative filtering algorithms. Deng et al. [14] proposed a K-medoids clustering recommendation algorithm based on the probability distribution for CF, which can effectively deal with the sparsity problem. Cao et al. [15] considered the influence of neighbor users and items, then proposed a unified probability matrix factorization recommendation algorithm to alleviate the sparsity of tag information and rating information. Zhang et al. [16] used trust relationship data of users to propose a recommendation algorithm based on the trust of users, which can effectively improve the accuracy of recommendations. He et al. [17] proposed a similarity measure model considering users’ preferences for item attributes. The model considers the user’s preferences for item attributes and co-rated items, and their results show that the number of co-rated items effectively improves the performance of the recommendation algorithm. Zhang et al. [18] introduced a method based on similarity measures and the corresponding collaborative filtering recommendation algorithm by introducing a common user item popularity rating between features and user relevance.

However, it is difficult to obtain sufficient social relations or label information in real data. In addition, in the above methods, it is generally assumed that the interaction relationship between users is fixed and bidirectional, but in real life, this assumption does not always stand. For example, if A is a fan of B, the behavior of B has a great impact on A, but not the inverse. In addition, it is worthwhile to mention that the interactions between users change gradually over time. Moreover, users’ preferences will change over time. Therefore, many scholars have recently considered temporal information in the recommendation system.

### 2.2. Collaborative Filtering Algorithm Considering Temporal Information

In order to increase CF accuracy, Vinagre et al. [19] introduced many algorithms that attempt to utilize temporal dynamics. Time-dependent algorithms treat time as a sequence and utilize time-dependent neighborhood models or factorization models to increase the algorithm’s capacity for prediction, whereas time-aware algorithms approach time as a context and use timestamps as an additional information source to enhance the model. Zhang et al. [20] proposed a new collaborative filtering recommendation algorithm based on probability matrix factorization, which integrates time weights into the rating matrix to construct a user-item-time model. Li et al. [21] proposed a personalized recommendation algorithm with social information and dynamic time windows, integrated social information and user interest in the process of searching the nearest neighbor, and allocated corresponding time weights for users’ interests in different periods. In order to improve the accuracy of the recommendation algorithm, Chen et al. [22] proposed an information backbone extraction method based on a personalized time window. Ren et al. [23] classified the user’s preference pattern into a sparse matrix according to the user’s preference pattern and the temporal characteristics of preferences, and then used the subspace to gradually model the individualization and the global aspects. Xiao et al. [24] proposed an improved clustering-based collaborative filtering recommendation algorithm in which a time decay function was introduced to preprocess users’ evaluation scores, the project attribute vector was used to represent the project, the user interest vector was used to describe the user, and the clustering algorithm was used to describe the cluster.

Although the approaches mentioned above have incorporated users’ relationships or temporal information into the algorithm design process, they rarely do so simultaneously. For this problem, our paper proposes a recommendation algorithm that takes into account both users’ relationships and temporal information. The main contributions of this paper are as follows: (1) constructs a personalized sliding time window for each user; calculates the similarity between different users through sequence alignment. (2) The algorithm only needs to extract part of the data in the original dataset, which improves the execution efficiency.

## 3. Materials and Methods

### 3.1. Recommended Method Based on Sequence Alignment

Zhang et al. [25] found that using all historical data of users does not improve the accuracy of recommendations and may even be misleading. By adopting certain strategies to remove redundant and worthless information and extract only part of the valuable key information required by the recommendation system, which is called the information backbone, not only the performance of the recommendation system can be improved, but also the execution efficiency of the algorithm can be improved. Based on this, this paper uses network topology information and temporal information to extract some key information to form the information backbone.

Differentiating the size of the information backbone by different users, we set different information backbone extraction schemes for different users. The basic idea of the algorithm is that we set a parameter according to the degree of each user in the system and then set different information backbone extraction schemes for users according to different parameters, and finally reconstruct the information backbone of all users into a complete bipartite graph. The new network replaces the original network in the computation.

We set the initial time of the sliding window to $t_{s}$, the current time is *t*, and the sequence of ratings of the user *u* in the sliding window is:

(1) $$\left(o_{u1},s_{u1},t_{u1}\right),\left(o_{u2},s_{u2},t_{u2}\right),\cdots ,\left(o_{um},s_{um},t_{um}\right)$$

where $t_{s}\leq t_{u1}\leq \cdots \leq t_{um}\leq t$, the *k* item $\left(o_{uk},s_{uk},t_{uk}\right)$ represents that user *u* at time $t_{uk}$ rate product $o_{uk}$, and the rating is $s_{uk}$.

If we assume $r_{ui}$ is the predicted rating of user *u* to rate item $o_{i}$ at the current time *t*, then $r_{ui}$ is calculated by:

(2) $$r_{ui}={\bar{r}}_{u}+\frac{\sum_{v\in p(i)}\left({\bar{r}}_{vi}-{\bar{r}}_{v}\right)sim_{uv}}{\sum_{v\in p(i)}sim_{uv}}$$

where *P(i)* is a collection of users who have rated at least one item $o_{uk}$ within the sliding window, $sim_{uv}$ is the similarity between users *u*, *v* in the sliding window, and ${\bar{r}}_{u}$ is the average of the user’s rating of all items in the sliding window, we use the following equation:

(3) $${\bar{r}}_{u}=\frac{\sum_{i=1}^{m}s_{ui}\alpha^{\left(t-t_{ui}\right)}}{\sum_{i=1}^{m}\alpha^{\left(t-t_{ui}\right)}}$$

where α∈(0,1) is the attenuation coefficient; it gives newer ratings a larger weight, while older ratings have less impact on the results.

In Formula (2), ${\bar{r}}_{vi}$ is the average value of the user’s rating of the item $o_{i}$ in the sliding window. We calculate it by:

(4) $${\bar{r}}_{vi}=\frac{\sum_{o_{vk}=o_{i}}s_{vk}\alpha^{\left(t-t_{vk}\right)}}{\sum_{o_{vk}=o_{i}}\alpha^{\left(t-t_{vk}\right)}}$$

The rating sequences of the users *u* and *v* in the sliding window are shown in (5) and (6), and their lengths are *n* and *l*, respectively.

(5) $$Q_{u}=\left(o_{u1},s_{u1},t_{u1}\right),\left(o_{u2},s_{u2},t_{u2}\right),\cdots ,\left(o_{um},s_{um},t_{um}\right)$$

(6) $$Q_{v}=\left(o_{v1},s_{v1},t_{v1}\right),\left(o_{v2},s_{v2},t_{v2}\right),\cdots ,\left(o_{vl},s_{vl},t_{vl}\right)$$

When calculating the similarity between users *u* and *v* in the sliding window, we should consider the timing relationship of the user’s evaluation of the products in the above two sequences. That is, not only the similarity of the ratings of the two users to the product but also the similarity of the time of their ratings. The closer the rating time to the same product, the higher the similarity. In addition, the similarity of their rating sequences should be considered, which means the similarity in the order of evaluation of different items. The more consistent the ranking of different products, the higher the similarity. Therefore, according to the ratings of users *u* and *v* in the sliding window in (5) and (6), we determine the similarity by finding the sub-sequences that match the most between them and propose a dynamic programming-based algorithm to calculate the similarity of the two sequences.

For the rating sequences of the lengths *m* and *l* of the users *u* and *v* in the sliding window in (5) and (6), we set their sub-sequences as *i* and *j*, respectively.

The similarity of $\left(o_{u1},s_{u1},t_{u1}\right),\cdots ,\left(o_{ui},s_{ui},t_{ui}\right)$ and $\left(o_{v1},s_{v1},t_{v1}\right),\cdots ,\left(o_{vj},s_{vj},t_{vj}\right)$ is $sim_{uv}\left(i,j\right)$, we set $sim_{uv}\left(i,0\right)=sim_{uv}\left(0,j\right)=0$, (*i* = 1, 2, ⋯, *m*; *j* = 1, 2, ⋯, *l*). If we assume $sim_{uv}\left(i,j-1\right)$ and $sim_{uv}\left(i-1,j\right)$ are known, then $sim_{uv}\left(i,j\right)$ can be obtained in the following:

(1) If $o_{ui}\neq o_{vj}$; that is, the product of the *i*-th rating of the user *u* is different from the item of the *j*-th rating of the user *v*, then

(7) $$sim_{uv}\left(i,j\right)=max\left\{sim_{uv}\left(i,j-1\right),sim_{uv}\left(i-1,j\right)\right\}$$

(2) If $o_{ui}=o_{vj}$; that is, the product of the i-th rating of the user *u* is the same as the item of the *j*-th rating of the user *v*, then

(8) $$sim_{uv}\left(i,j\right)=max\left\{sim_{uv}\left(i,j-1\right)+\Delta_{ij},sim_{uv}\left(i-1,j\right)+\Delta_{ij}\right\}$$

where $\Delta_{ij}$ is the increment of the similarity of the product of the *i*-th rating of user *u* and the *j*-th rating of user *v*, which can be calculated by the following formula:

(9) $$\Delta_{ij}=\left[1-\frac{|s_{ui}-s_{vj}|}{max(s_{ui},s_{vj})}\right]\alpha^{t_{ui}-t_{vj}}$$

When calculating the increment of similarity between users *u* and *v* in (9), the same degree of rating the same product is considered, and the closeness of their rating time is considered. The first part of (9) is the same degree of rating, as measured by the relative error of the rating. The second part of (9) is the closeness of their rating time, which is adjusted by the attenuation coefficient. The longer the rating interval, the greater the attenuation of the similarity value [20]. We calculate the value of $sim_{uv}\left(i,j\right)$ according to the above rules and finally obtain the value of $sim_{uv}\left(m,l\right)$, which is the similarity between users *u* and *v*.

### 3.2. The Framework of the Similarity Algorithm

The framework of our similarity algorithm based on dynamically planned sequences is as follows (Algorithm 1):
**Algorithm 1:** Sequence_sim(*u*,*v*).**Input:** input $Q_{u}$, $Q_{v}$: the rating sequence of users *u* and *v***Output:** output $sim_{uv}$: the similarity between users *u* and *v*    1:Begin    2:Set $sim_{uv}\left(i,0\right)=sim_{uv}\left(0,j\right)=0,\left(i=1,2,\ldots ,m;j=1,2,..,l\right)$    3:**for***i=1 to m***do**    4:      **for** *j=1 to l* **do**    5:            **if** $o_{ui}\neq o_{vj}$ **then**    6:                  $sim_{uv}\left(i,j\right)=max\left\{sim_{uv}\left(i,j-1\right),sim_{uv}\left(i-1,j\right)\right\}$    7:            **else**    8:                  $sim_{uv}\left(i,j\right)=max\left\{sim_{uv}\left(i,j-1\right)+\Delta_{ij},sim_{uv}\left(i-1,j\right)+\Delta_{ij}\right\}$    9:            **end if**  10:      **end for**  11:**end for**  12:$sim_{uv}=sim_{uv}\left(m,l\right)$  13:End

In this algorithm, m is the size of the extracted information backbone of user *u*, and *l* is the size of the extracted information backbone of user *v*. We define a list object sim in the algorithm to save the similarity information of users u and v in the time window. We set the initial value $sim_{uv}\left(i,0\right)=sim_{uv}\left(0,j\right)=0$, where i=1,2,...,m, and j=1,2,...,l, the similarity value of each subsequent element in sim can be calculated from the values of the previous elements by selecting different formulas according to the conditions, and its last element value is the final calculated similarity of user u and v. Obviously the algorithm is a double loop, and the algorithm time complexity is O(m∗l). Since both *m* and *l* are the sizes of extracting part of the data in the dataset, the execution efficiency of the algorithm will be significantly improved compared to extracting all of the data.

### 3.3. Personalized Recommendation Algorithm Based on Sliding Window

Let $S=[s_{ij}]$ be a rating matrix, where the value of element $s_{ij}$ is the rating sequence of user *i* for item *j*. When the value of element $s_{ij}$ is empty, it means that user *i* does not rate item *j*. Let $T=[t_{ij}]$ be the rating time matrix. When the value of element $s_{ij}$ of the rating matrix S is not empty, the value of the corresponding element $t_{ij}$ of T is not empty, which is the time series of user *i*’s rating of item *j*. Taking the *i*-th row of the matrices *S* and *T*, it is easy to construct a rating sequence of user *i* in the form of (5).

Our framework for personalized recommendation algorithms based on sliding windows is as follows (Algorithm 2):
**Algorithm 2:** SSWT (Personalized recommendation algorithm based on sliding window)**Input:** S: Rating Matrix; T: Rating time matrix;**Output:** R: Recommendation matrix;**Begin:****Step1:** Set a value for each target user based on the user’s degree. Its calculation formula is (19):(10)$\theta_{i}=\frac{k_{ui}-min\left(k_{u}\right)}{max\left(k_{u}\right)-min\left(k_{u}\right)}$
Here, the degree of the user refers to the number of different commodities purchased by the user, and $k_{ui}$ denotes the degree of the *i*-th user. If a user’s degree is larger, it means that the user is more active and the time window spans a shorter time period; on the contrary, if a user’s degree is small, it means that the user has little historical data and the time window selected spans a longer time period. In determining the sliding time window for each user, it is necessary to select different time ranges according to the user’s degree. Parameter $\theta_{i}$ in Formula (10) is calculated according to the user’s degree, and its value is between 0 and 1. The greater the user’s degree, the greater the value of $\theta_{i}$, and vice versa.**Step2:** We calculate the initial time of the corresponding sliding window for each user:(11)$$TS_{ui}=TE\times \alpha \log_{2}\left(\theta_{i}+\beta \right)$$
where $TS_{ui}$ is user *i* at the initial time of the training set, *TE* is the termination time of the training set, and α and β are parameters. The greater parameter $\theta_{i}$, the more active the user is and the closer the initial time of the time window is to the end of the dataset. The values of parameters α and β will be determined in the experiment according to the experimental results.**Step3:** Each user has a time window of the training set, and the size of the window is: [*TS*, …, *TE*](*i* = 1, 2,..., *N*), where *N* is the number of users. According to the time window, the rating matrix *S*, and the rating time matrix *T*, the rating sequence $Q_{u}$ of each user *u* in the time window is determined, and all the user rating sequences are used as the training set of the algorithm; that is, the information backbone.**Step4:** For each *u* and *v*:We call the algorithm Sequence_sim(u,v) to calculate their similarity $sim_{uv}$.**Step5:** For each user *u*:We calculate the mean of all products rating in the sliding window by ${\bar{r}}_{u}$.**Step6:** For each user *u* and product $o_{i}$:We calculate the mean value ${\bar{r}}_{ui}$ of product $o_{i}$’s rating in the sliding window according to Formula (4).**Step7:** For each user *u* and product $o_{i}$:We calculate the predicted rating $r_{ui}$ of product $o_{i}$ at the current time *t* by user *u* according to Formula (2); We generate a rating matrix $R=[r_{ui}]$ as the final rating matrix.**Step8:** The recommended list of users in each row of *R* is sorted from largest to smallest to form a recommendation result.**End**

**Figure 1:**
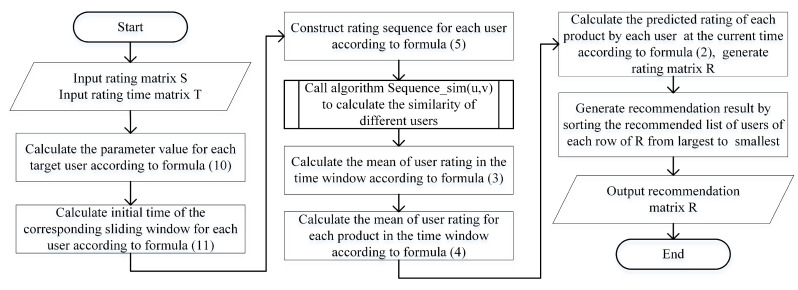
Flowchart of the SSWT algorithm.

The algorithm flowchart is shown in Figure 1.

In the first step of the algorithm, we set a parameter for each user according to the degree of each user. The greater the degree, the larger the value. Through the second step of the algorithm, we can see that the time window of the large user is relatively small, and the time window of the small user is relatively large. Then, when the information backbone is extracted, the information of a small user is larger than that of a large user. The backbone is relatively large, indicating that we need to use the long-term historical information of the small users when recommending, which avoids the cold start problem caused by the extraction of the information backbone from the small users. For large users, because they purchase goods more frequently, long-term purchase history information will cause misleading recommendations, so we only need their short-term historical purchase information as the information backbone, so the algorithm sets personalized information backbone extraction schemes for different users.

When the sliding window changes over time, the algorithm does not need to recalculate the user similarity and rating values for the new window. It only needs to incrementally modify the results according to the newly added rating on the result of the previous window.

## 4. Experiment and Discussion

### 4.1. Experimental Datasets

Three standard datasets are applied in this study to evaluate the performance of recommendation algorithms. The first dataset is the sampling of the four months of data from February 2001 to May 2001 from the Netflix website (http://www.Netflixprize.com/, accessed on 10 July 2020). The movies are rated from 1 (worst) to 5 (best) by the users. A total of 419,247 links were obtained from the rating filtering process. The second dataset is the sampling of the three years of data from January 2002 to May 2005 from the Movielens website (http://www.grouplens.org/, accessed on 20 May 2020). The dataset consists of 4999 users and 7471 movies. Similarly, the Movielens dataset is based on 5-star evaluation, and we extracted 730,156 links from this website. The third dataset is Epinion, which contains 664,824 rating records for 139,738 items, and each item has been rated at least once by 49,290 users. The Epinion dataset also contains the trust relationship between users, and there are 487,181 user trust pairs in total.

### 4.2. Evaluation Indicators

The current common indicators for measuring the accuracy of link prediction algorithms are Precision and Ranking Score [26]. Moreover, Diversity and Popularity [27] are often used in personalized recommendation predictions for bipartite networks. The main evaluation indicators are as follows:

(1) Precision

Precision only considers whether the edge of the top L in the target user’s rating list is predicted to be accurate. If there are m predictions that are accurate, i.e., m of the edges of the top L is in the test set, Precision is defined as:

(12) $$precision=\frac{m}{L}$$

Obviously, the higher the Precision value, the higher the accuracy of the algorithm.

(2) Ranking Score

The main consideration is the position of the edges in the test set in the final ranking. Define G = (V,E) as an undirected network, where V is the set of nodes and E is the set of edges. The total number of nodes in the network is N, and the number of edges is M. The network has a total of N*(N − 1)/2 node pairs, which make up the set U. The known edges E are divided into two parts: training set $E^{T}$ and test set $E^{P}$.

Let H = U−$E^{T}$ be the set of unknown edges, $r_{i}$ represents the ranking in all i $\in E^{P}$. Then the Ranking Score value of the unknown edge is $RS_{i}=r_{i}/\left|H\right|$, where |H| represents the number of elements in set H. We traverse all edges in the test set to get the system’s Ranking Score value:

(13) $$RS=\frac{1}{|E^{P}|}\sum_{i\in E^{P}}RS_{i}=\frac{1}{|E^{P}|}\sum_{i\in E^{P}}\frac{r_{i}}{\left|H\right|}$$

(3) Popularity

In the process of personalized recommendation of the bipartite network, recommending non-popular products to users is more meaningful than recommending popular products. Popularity is an indicator used to evaluate the novelty of a recommendation. In fact, it is to calculate the average value of all the items in each user’s recommendation list. The user’s corresponding Popularity calculation formula is as follows:

(14) $$p_{i}=\frac{1}{L}\sum_{\alpha =1}^{L}k_{\alpha }$$

where $k_{\alpha }$ is the degree of product $o_{\alpha }$, and L is the length of the recommendation list. Here, $k_{\alpha }$ indicates the value of the Popularity of a product $o_{\alpha }$ in the recommendation list. The greater the degree of a product, the more frequently the product is purchased. If a recommendation algorithm has a high Popularity index, it means that the algorithm is more likely to recommend popular things, while the lower the value, it means that the algorithm is more likely to recommend unpopular personalized products. The Popularity of the entire recommendation system is the average of all user Popularity, and the calculation formula is as follows:

(15) $$p=\frac{1}{n}\sum_{i=1}^{L}p_{i}$$

We can see that the lower the Popularity of the recommendation algorithm, the better the recommended effect.

(4) Diversity

Recommendation diversity is an important indicator for evaluating the performance of bipartite networks. A good recommendation system can recommend different types of products according to the different characteristics of users. The more similar a recommendation system is for a user, the less effective the recommendation system is. Two different indicators can be used to assess the diversity of the system, which are inter-user diversity and intra-user diversity.

Inter-user diversity is also known as the Hamming distance; it is an indicator for evaluating the similarity between different user recommendation lists. The formula for calculating the diversity between any two users is as follows:

(16) $$H_{ij}=1-\frac{Q}{L}$$

where L is the length of the recommendation list, Q is the number of the same items in user $u_{i}$ and $u_{j}$ recommendation lists. The smaller the number of the same item, the larger the Hamming distance, and the mutual diversity calculation formula of the whole system is as follows:

(17) $$D_{inter}=\frac{1}{n(n-1)}\sum_{i=1}^{n}\sum_{j=i+1}^{n}H_{ij}$$

The greater the value of inter-user diversity, the better the diversity of the system’s recommendations.

Intra-user diversity is an indicator for evaluating the similarity between all the users. For any user $u_{i}$, the corresponding self-diversity calculation formula is as follows:

(18) $$D_{intra}^{i}=\frac{1}{L(L-1)}\sum_{\alpha \neq \beta }\left(1-s_{\alpha \beta }\right)$$

where $s_{\alpha \beta }$ is the similarity of product $o_{\alpha }$ and $o_{\beta }$ in $u_{i}$, it is calculated by:

(19) $$s_{\alpha \beta }=\frac{1}{\sqrt{k_{\alpha }k_{\beta }}}\sum_{i=1}^{n}a_{i\alpha }a_{i\beta }$$

where $k_{\alpha }$ and $k_{\beta }$ are the degrees of $o_{\alpha }$ and $o_{\beta }$. Then the self-diversity calculation formula of the entire recommendation system is as follows:

(20) $$D_{intra}=\frac{1}{n}\sum_{i=1}^{n}D_{intra}^{i}$$

If the similarity of the items in each user recommendation’s list is smaller, the system’s self-diversity value is greater.

### 4.3. Experiment and Results

**Figure 2:**
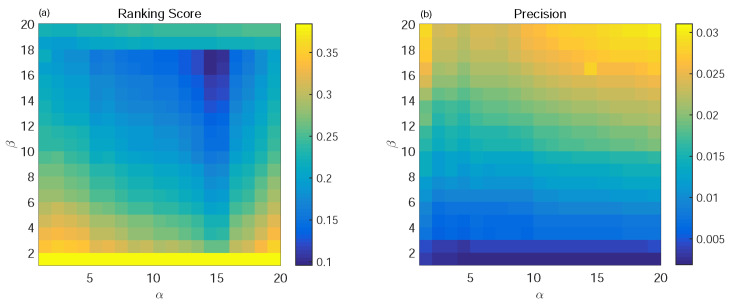
The variation tendencies of Ranking Score (**a**) and Precision (**b**) with α and β on the Netflix dataset.

**Figure 3:**
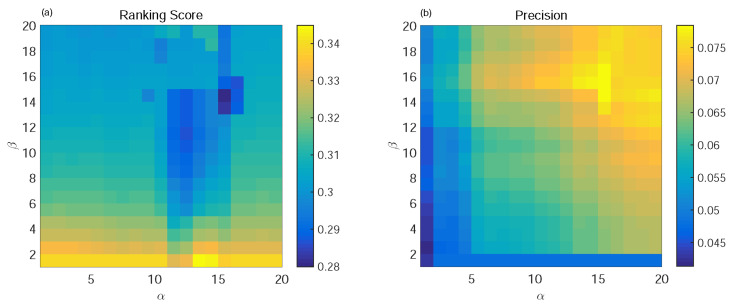
The variation tendencies of Ranking Score (**a**) and Precision (**b**) with α and β on the Movielens dataset.

In the process of user selection of personalized window length, we know from (19) that the larger the user’s degree, the larger the *TS* value, and the shorter the length of the sliding window. Such properties indicate that we only need to use a small part of the information from the user as their information backbone. On the contrary, since the information of small users is relatively scattered, we need to expand the size of the sliding window for the extraction of the information backbone. We denote by α and β the parameters to adjust the size of the sliding window; we set α∈[0,1]. Figure 2 and Figure 3 show the performance changes of four indexes in two datasets under different α and β.

From Figure 2 and Figure 3, we can see that when α = 0, which means *TS* = 0 and all of the information was taken into the information backbone network, then the value of the corresponding metrics obtained are not impressive. We use the whole dataset as the training set for the recommendation system, and maybe there is some redundant information to interfere with the design of the recommendation system. However, we can see that a larger α means a larger β. On the contrary, if the information backbones selected by the user are the smallest, the performance of the recommendation system is not optimal because, at this time, the information backbones of both large and small users contain less information, and some important information may be lost. Therefore, we finally selected α and β as suitable for different datasets as the optimal parameters.

In the SSWT algorithm, we set α = 0.7 and β = 0.8 for Netflix and α = 0.75 and β = 0.7 for Movielens. We compared the traditional global ranking algorithm (GRM) [28], user-based collaborative filtering algorithm (UCF), mass diffusion algorithm (MD) [29], and time SVD++ [30]. In the experiment, the ratio of the training set is 0.9, and the four indices of the evaluation metrics are Ranking Score, Precision, Popularity, and Diversity. The best results are shown in bold. The experimental results are shown in Table 1.

### 4.4. Discussion

Accuracy has always been the primary indicator for recommendations. As can be seen from Table 1, in the Netflix dataset, the Ranking Score value of the SSWT algorithm is reduced by 16.81%, 18.45%, 11.49%, and 7.19%, respectively, relative to the GRM, UCF, MD, and time SVD++ algorithms. The Precision value is increased by 68.59%, 37.02%, 21.05%, and 8.05%, respectively, relative to the four algorithms. In the Movielens dataset, the SSWT algorithm’s Ranking Score is reduced by 35.63%, 25.34%, 24.69%, and 16.15%, respectively, relative to the GRM, UCF, MD, and time SVD++ algorithms. The Precision value is increased by 56.40%, 38.20%, 37.97%, and 15.64%, respectively, relative to the other algorithms. Therefore, the accuracy of the SSWT algorithm is better than the other four algorithms. In the Epinion dataset, the Ranking Score value of the SSWT algorithm is reduced by 18.98%, 13.27%, 10.31%, and 5.22%, respectively, relative to the GRM, UCF, MD, and time SVD++ algorithms. The Precision value is increased by 62.68%, 29.98%, 25.90%, and 12.32%, respectively, relative to the four algorithms.

Diversity and Popularity are also important indicators for measuring the effectiveness of the two-point network recommendation. As can be seen from Table 1, the average Popularity of the SSWT algorithm in the three datasets is lower than the other four algorithms. In the Netflix dataset, the Popularity value of the SSWT algorithm is reduced by 19.09%, 12.42%, 7.05%, and 4.01%, respectively, relative to the GRM, UCF, MD, and time SVD++ algorithms. The Diversity value of the SSWT algorithm increased by 105.81%, 27.66%, 6.31%, and 3.78%, respectively, relative to the four algorithms. In the Movielens dataset, the Popularity value of the SSWT algorithm is reduced by 27.42%, 26.29%, 26.13%, and 16.02%, respectively, relative to the GRM, UCF, MD, and time SVD++ algorithms. The Diversity value of the SSWT algorithm increased by 25.31%, 9.14%, 6.28%, and 4.76%, respectively, relative to the four algorithms. In the Epinion dataset, the Popularity value of the SSWT algorithm is reduced by 19.12%, 16.89%, 14.18%, and 7.20%, respectively, relative to the GRM, UCF, MD, and time SVD++ algorithms. The Diversity value of the PR-SW algorithm increased by 52.19%, 41.81%, 20.10%, and 7.99%, respectively, relative to the four algorithms.

From the experiment, it can be concluded that in the Netflix and Movielens datasets, the number of links in the information backbone only accounted for 46.3% and 46.7% of the original network. This shows that the SSWT algorithm can not only remove a large amount of redundant information in the backbone extraction process but also reduce the storage space of the network.

## 5. Conclusions

This paper mainly discusses the phenomenon of information redundancy in the recommendation process of the network. We use the personalized time window to predict and recommend the network, set different parameters for different target users, and pass different users in the time window. The time series scores are compared to form similarities between users. Finally, the user’s recommendation list is constructed according to the similarity between users. The experimental results show that the time window-based recommendation algorithm can not only delete a large amount of redundant information in the data core but also effectively improve the performance of the personalized recommendation algorithm.

## Figures and Tables

**Table 1:** Performance of the five algorithms.

Dataset	Indicator	GRM	UCF	MD	Time SVD++	SSWT
Netflix	RS	0.1148	0.1171	0.1079	0.1029	**0.0955**
	Precision	0.0191	0.0235	0.0266	0.0298	**0.0322**
	Popularity	1687.7	1559.1	1469.0	1422.6	**1365.5**
	Diversity	0.3391	0.5467	0.6565	0.6725	**0.6979**
Movielens	RS	0.3469	0.2991	0.2965	0.2663	**0.2233**
	Precision	0.0539	0.0610	0.0611	0.0729	**0.0843**
	Popularity	1453.5	1431.3	1428.1	1256.2	**1055.0**
	Diversity	0.5730	0.6579	0.6756	0.6854	**0.7180**
Epinion	RS	0.2534	0.2367	0.2289	0.2166	**0.2053**
	Precision	0.0493	0.0617	0.0637	0.0714	**0.0802**
	Popularity	1568.4	1526.3	1478.1	1366.9	**1268.5**
	Diversity	0.4629	0.4968	0.5866	0.6524	**0.7045**

## Data Availability

All data are presented in main text.
